# Dynamical behaviors of a stochastic SIVS epidemic model with the Ornstein-Uhlenbeck process and vaccination of newborns

**DOI:** 10.1371/journal.pone.0310175

**Published:** 2024-10-23

**Authors:** Shenxing Li, Wenhe Li

**Affiliations:** School of Mathematics and Statistics, Northeast Petroleum University, Daqing, Heilongjiang, China; Adana Alparslan Türkeş Science and Technology University: Adana Alparslan Turkes Bilim ve Teknoloji Universitesi, TÜRKIYE

## Abstract

In this paper, we study a stochastic SIVS infectious disease model with the Ornstein-Uhlenbeck process and newborns with vaccination. First, we demonstrate the theoretical existence of a unique global positive solution in accordance with this model. Second, adequate conditions are inferred for the infectious disease to die out and persist. Then, by classic Lynapunov function method, the stochastic model is allowed to obtain the sufficient condition so that the stochastic model has a stationary distribution represents illness persistence in the absence of endemic equilibrium. Calculating the associated Fokker-Planck equations yields the precise expression of the probability density function for the linearized system surrounding the quasi-endemic equilibrium. In the end, the theoretical findings are shown by numerical simulations.

## 1 Introduction

Epidemic models with vaccination are one of the important models for studying epidemiology. The purpose of epidemiological research is to constrain and avoid disease incidence and transmission. Protection of susceptible population is one of the important means of preventing and dominating infectious diseases, and vaccination is an effective measure to protect susceptible population. Taking vaccination into account, Zhao et al. [1] formulated a stochastic SIS model with vaccination, giving a threshold for the model when the white noise intensity is low. Zhang et al. [2] studied a stochastic SVIR model and found that white noise contributed to disease control. Considering more realistic factors, many models of infectious diseases with vaccination have been explored [3–6].

However, vaccination of the population does not mean permanent immunity. After a while, they may lose their immunity. This phenomenon has been studied in many literatures [7–9]. In Ref. [10], authors studied an SIS infectious disease model with vaccination and changing total population size, where the effectiveness of vaccines is time-limited. In Ref. [11], the following SIS model with immunization was also discussed by Li and Ma,

$$\begin{array}{c} \left\{ \begin{array}{cc} \text{d}S= & [(1-g)A-\beta SI-(\eta +p)S+\gamma I+\alpha V]\text{d}t,\\ \text{d}I= & [\beta SI-(\eta +\gamma +\varepsilon )I]\text{d}t,\\ \text{d}V= & [Ag+pS-(\eta +\alpha )V]\text{d}t, \end{array}\right. \end{array}$$ (1)

where *S*(*t*), *I*(*t*) and *V*(*t*) are individuals who are susceptible to infection, infection, and vaccination, respectively. *g*(0 < *g* < 1) is a proportion of newborn vaccinations, *A* is a constant growth in the population, *η* is the natural mortality rate for these three compartments, *p* is the proportion of vulnerable individuals with vaccination, *β* is the coefficient of transmission between *S* and *I*, *γ* is the recovery rate of infectious individuals, *α* is the proportion of vaccinated people who have lost immunity, *ε* is the cause-specific mortality of infectious individuals. The lethality of the disease is less than the proportion of vulnerable individuals with vaccination, based on the realities of the social environment. Assume that all parameters are non-negative and *η*, *A* > 0.

By studying model Eq (1), Li et al. [11] obtained several important conclusions as follows:

(1) The threshold is $R_{0}=\frac{\beta A[\eta (1-g)+\alpha ]}{\eta (\eta +\gamma +\varepsilon )\left(\eta +\alpha +p\right)}$,
(2) The system exists the disease-free equilibrium $P_{0}=(S_{0},I_{0},V_{0})=(\frac{A[\eta (1-g)+\alpha ]}{\eta (\eta +\alpha +p)},0,\frac{A(\eta g+p)}{\eta (\eta +\alpha +p)})$ and the endemic equilibrium P* = (S*, I*, V*), where
  $$\begin{array}{c} S^{*}=\frac{\eta +\gamma +\varepsilon }{\beta },\;I^{*}=\frac{\eta (\eta +\gamma +\varepsilon )(\eta +\alpha +p)}{\beta (\eta +\varepsilon )(\eta +\alpha )}\left(R_{0}-1\right),\;V^{*}=\frac{gA\beta +p(\eta +\gamma +\varepsilon )}{\beta (\eta +\alpha )}, \end{array}$$
(3) *P*_0_ is globally asymptotically stable when *R*_0_ < 1, P* is globally asymptotically stable when *R*_0_ > 1.

In fact, environmental fluctuations inevitably affect the occurrence and development of infectious diseases. Considering infectious diseases as a stochastic process is therefore more relevant. For this reason, Beddington and May [12] proposed the parameter perturbation method for the first time, which is a classical method to derive stochastic differential equation model from the corresponding deterministic system, and this method has been widely used [13–17]. Liu et al. [18] investigated a stochastic SIQR infectious disease model that introduced the effects of two types of environmental noise, namely white noise and telegraph noise. They deduced adequate conditions for *I*(*t*) to persist and become extinct. There are sufficient conditions to prove that a positive recurrence solution exists when *I*(*t*) persists. Teng et al. [19] studied a stochastic SIS model with non-linear incidence, where white noise interferes with the propagation coefficient *β*. They obtained the threshold *R*_0_ for determining disappearance and prevalence of disease. In order to learn disease dynamics in a random environment, the next SIVS model was studied by Zhao et al. [20],

$$\begin{array}{c} \left\{ \begin{array}{cc} \text{d}S= & [(1-g)A-\beta SI-(\eta +p)S+\gamma I+\alpha V]\text{d}t-\theta SI\text{d}B(t),\\ \text{d}I= & [\beta SI-(\eta +\gamma +\varepsilon )I]\text{d}t+\theta SI\text{d}B(t),\\ \text{d}V= & [Ag+pS-(\eta +\alpha )V]\text{d}t. \end{array}\right. \end{array}$$ (2)

Clearly, the model Eq (2) is randomly derived from the deterministic model Eq (1) via

$$\begin{array}{c} \beta (t)=\beta +\theta \text{d}B(t). \end{array}$$

*B*(*t*) is the standard Brownian movement, *θ* is the intensity of white noise. After integrating both sides and dividing by *t*, we get

$$\begin{array}{c} \frac{1}{t}\int_{0}^{t}\beta (s)\text{d}s=\beta +\theta \frac{\text{B}(\text{t})}{\text{t}}∼N\left(\beta ,\frac{\theta^{2}}{t}\right). \end{array}$$

It’s easy to get Var[β(t)]→∞ as *t* → 0. This suggests that the fluctuations of *β*(*t*) may become very large when the time interval is very small, which is clearly implausible. In Ref. [21], Allen indicated that compared with the linear function of white noise, the mean-reverting process has an advantage in showing the diversity of the environment. The mean-reversion process has a wide range of applications in both finance and physics [22–24]. The parameters are set by a linear function of white Gaussian noise when they are perturbed by white Gaussian noise. However, there is another approach where the parameters are associated with a mean-reversion process [25–30], namely the Ornstein-Uhlenbeck process, in the form of:

$$\begin{array}{c} \beta =\beta +m(t), \end{array}$$

where *m*(*t*) satisfies

$$\begin{array}{c} \text{d}m(t)=-km(t)\text{d}t+\theta \text{d}B(t). \end{array}$$ (3)

Notice that *k*, *θ* > 0. *k* is the reversion speed, *θ* is the volatility intensity of process *m*(*t*), *B*(*t*) represents a standard Brownian movement. By simple calculations, we can obtain $E[m(t)]=m_{0}e^{-kt},Var[m(t)]=\frac{\theta^{2}}{2k}(1-e^{-2kt})$, where *m*_0_ ≔ *m*(0). Unlike white Gaussian noise, Var[β(t)] with the Ornstein-Uhlenbeck process tends to 0 as *t* → 0. Moreover, according to Ref. [31–33], it is easy to get *m*(*t*) to be ergodic and to converge weakly to the invariant density

$$\begin{array}{c} \zeta \left(x\right)=\frac{\sqrt{k}}{\sqrt{\pi }\theta }e^{-\frac{kx^{2}}{\theta^{2}}},\;\left(x\in R\right). \end{array}$$

So, using the ergodic theorem in Ref. [34], one obtains

$$\begin{array}{c} \underset{t\to \infty }{\text{lim}}\frac{1}{t}\int_{0}^{t}|m(\tau )|\text{d}\tau =\int_{-\infty }^{+\infty }|x|\zeta (x)\text{d}x=\frac{\theta }{\sqrt{\pi k}}. \end{array}$$ (4)

Then, for a sufficiently small time interval Δ*t*, the correlation coefficient of the Ornstein-Uhlenbeck process *m*(*t*) is *r*(*m*(*t*), *m*(*t* + Δ*t*)) = 1 − *o*(Δ*t*) and *r*(*m*(*t*), *m*(*t* + Δ*t*)) = 0 for white Gaussian noise [21]. Since the correlation coefficient between neighbouring states is close to 1, this indicates a high degree of correlation between neighbouring states in the Ornstein-Uhlenbeck process. This allows the model to better capture the transmission direction and related structure of epidemics. In contrast, white Gaussian noise has a correlation coefficient close to 0, which does not reflect the correlation of infectious diseases.

At the same time, there are many interacting variables in the environment that affect the infectious disease system, and these variables change in a continuous manner. In contrast to white Gaussian noise, the Ornstein-Uhlenbeck process is continuous (namely, the sample paths are continuous functions), which makes the model more realistic and interpretable and better reflects the continuous nature of infectious diseases. In summary of the comparison, we choose to introduce the Ornstein-Uhlenbeck process instead of white Gaussian noise.

Combining with the Eqs (1) and (3), the stochastic model is as follows:

$$\begin{array}{c} \left\{ \begin{array}{cc} \text{d}S= & [(1-g)A-\beta SI-mSI-(\eta +p)S+\gamma I+\alpha V]\text{d}t,\\ \text{d}I= & [\beta SI+mSI-(\eta +\gamma +\varepsilon )I]\text{d}t,\\ \text{d}V= & [Ag+pS-(\eta +\alpha )V]\text{d}t,\\ \text{d}m= & -km\text{d}t+\theta \text{d}B(t). \end{array}\right. \end{array}$$ (5)

Assume that $\left(\Omega ,\{F_{t}{\}}_{t\geq 0},P\right)$ is a complete probability space with a filtration {*F*_*t*_}_*t*≥0_ that meets the usual conditions (it is right continuous and *F*_0_ includes all P-null sets).

The paper is organised below. In Section 2, the existence of a unique positive solution for the system Eq (5) is proved. We obtain the condition $R_{0}^{S}$ that determines whether the disease will become extinct or persistent in Section 3. When $R_{0}^{S}>1$, the disease may persist; when $R_{0}^{S}<1$, the disease may become extinct. In Section 4, we attain the adequate requirement that can be prove the existence of the stationary distribution by composing appropriate Lyapunov functions. In Section 5, the precise expression for the density function of the linearised system correspondent to the stochastic system Eq (5) surrounding the quasi-equilibrium is derived. In Section 6, the validity of theoretical results will be demonstrated by means of numerical simulations.

## 2 Existence and uniqueness of the global solution

In analysing the dynamic behaviour of epidemic, considering whether the positive solution is global is a crucial step. In this section, we demonstrate the existence of the unique global positive solution.

**Theorem 2.1**. *For any initial value* $(S(0),I(0),V(0),m(0))\in {\mathbb{R}}_{+}^{3}\times \mathbb{R}$, *on t* ≥ 0, *there does exist a unique solution* (*S*(*t*), *I*(*t*), *V*(*t*), *m*(*t*)) *to system* Eq (5) *which stays with probability 1 in* $R_{+}^{3}\times R$.

**Proof**. Due to the local Lipschitz continuity of the coefficients of model Eq (5), there exists a unique local solution $(S(t),I(t),V(t),m(t))\in R_{+}^{3}\times R$ on *t* ∈ [0, *τ*_*e*_), where *τ*_*e*_ denotes the explosion time [35].

Next we verify that *τ*_*e*_ = ∞ a.s., with the aim of certificating that the solution is global. Make *r*_0_ > 0 sufficiently large so that *S*(0), *I*(0), *V*(0) and *e*^*m*(0)^ are in $\left[\frac{1}{r_{0}},r_{0}\right]$. For each integer *r* > *r*_0_, define the stopping time as

$$\begin{array}{c} \tau_{r}=\text{inf}\left\{t\in \left[0,\tau_{e}\right):\text{min}\left\{S\left(t\right),I\left(t\right),V\left(t\right),e^{m(t)}\right\}\leq \frac{1}{r}\;\text{or}\;\text{max}\left\{S\left(t\right),I\left(t\right),V\left(t\right),e^{m(t)}\right\}\geq r\right\}. \end{array}$$

Here we define inf ∅ = ∞ in the paper where ∅ is the empty set. As *r* → ∞, *τ*_*r*_ increases monotonically. Make $\tau_{\infty }=\underset{r\to \infty }{\text{lim}}\;\tau_{r}$, hence *τ*_∞_ ≤ *τ*_*e*_ a.s.. When *τ*_∞_ = ∞ a.s. is satisfied, then *τ*_*e*_ = ∞ a.s. and (*S*(*t*), $I(t),V(t),m(t))\in R_{+}^{3}\times R$ a.s., on *t* ≥ 0.

Then, consider the paradox, that is *τ*_∞_ < ∞ a.s., then there are the constants *h* > 0 and *δ* ∈ (0, 1) that make $P\{\tau_{\infty }\leq h\}>\delta$. Therefore, suppose that an integer *r*_1_ ≥ *r*_0_ makes $P\{\tau_{r}\leq h\}\geq \delta$ a.s., ∀*r* ≥ *r*_1_. Define the *C*^2^-function *W*_1_: $R_{+}^{3}\times R\to R_{+}$ as follows:

$$\begin{array}{c} W_{1}\left(S,I,V,m\right)=\left(S-1-\text{ln}\;S\right)+\left(I-1-\text{ln}\;I\right)+\left(V-1-\text{ln}\;V\right)+\frac{m^{4}}{4}. \end{array}$$

Using *u* − 1 ≥ ln *u* for all *u* > 0, it can be seen that the function is non-negative. From equation Eq (5), it is evident that the total population is regulated by

$$\begin{array}{c} \text{d}(S+I+V)=[A-\eta (S+I+V)-\varepsilon I]\text{d}t\leq [A-\eta (S+I+V)]\text{d}t, \end{array}$$

then

$$\begin{array}{c} S\left(t\right)+I\left(t\right)+V\left(t\right)\leq \{\begin{array}{cc} & \frac{A}{\eta },\\ & S(0)+I(0)+V(0), \end{array}\begin{array}{c} S\left(0\right)+I\left(0\right)+V\left(0\right)\leq \frac{A}{\eta },\\ S\left(0\right)+I\left(0\right)+V\left(0\right)>\frac{A}{\eta }, \end{array}. \end{array}$$

Suppose that $M=\text{max}\{S(0)+I(0)+V(0),\frac{A}{\eta }\}$. For ∀*r* ≥ *r*_0_ and ∀*h* > 0, *W*_1_ uses the Itô’s formula to produce:

$$\begin{array}{c} \text{d}W_{1}\left(S,I,V,m\right)=LW_{1}\text{d}t+m^{3}\theta \text{d}B\left(t\right), \end{array}$$ (6)

where

$$\begin{array}{c} \begin{array}{cc} LW_{1}= & \left(1-\frac{1}{S}\right)\left[A\left(1-g\right)-\left(\beta +m\right)SI-\left(\eta +p\right)S+\gamma I+\alpha V\right]+\left(1-\frac{1}{I}\right)[\left(\beta +m\right)SI\\ & -\left(\eta +\gamma +\varepsilon \right)I]+\left(1-\frac{1}{V}\right)\left[Ag+pS-\left(\eta +\alpha \right)V\right]-km^{4}+\frac{3}{2}\theta^{2}m^{2}\\ \leq & A+\left(\gamma +\beta \right)I+\alpha V+pS+m\left(I-S\right)+\left(3\eta +p+\gamma +\varepsilon +\alpha \right)-km^{4}+\frac{3}{2}\theta^{2}m^{2}\\ \leq & A+3\;\text{max}\left\{\gamma +\beta ,\alpha ,p\right\}M+\left(3\eta +p+\gamma +\varepsilon +\alpha \right)+\underset{m\in R}{\text{sup}}(-km^{4}+\frac{3}{2}\theta^{2}m^{2}\\ & +2|m|M)\\ \leq & k_{1}. \end{array} \end{array}$$

Here *k*_1_ > 0, and *k*_1_ is independent of the initial value. Substituting the above inequality into Eq (6) to obtain

$$\begin{array}{c} \text{d}W_{1}\left(S,I,V,m\right)\leq k_{1}\text{d}t+m^{3}\theta \text{d}B\left(t\right). \end{array}$$

Integrating from 0 to *τ*_*r*_ ∧ *h* and taking expectations, there is

$$\begin{array}{c} \begin{array}{cc} E[W_{1}\left(S\left(\tau_{r}\wedge h\right),I\left(\tau_{r}\wedge h\right),V\left(\tau_{r}\wedge h\right),m\left(\tau_{r}\wedge h\right)\right)] & \leq W_{1}\left(S\left(0\right),I\left(0\right),V\left(0\right),m\left(0\right)\right)+k_{1}E\left[\left(\tau_{r}\wedge h\right)\right]\\ & \leq W_{1}\left(S\left(0\right),I\left(0\right),V\left(0\right),m\left(0\right)\right)+k_{1}h. \end{array} \end{array}$$

Let Ω_*r*_ = {*τ*_*r*_ ≤ *h*} for *r* ≥ *r*_1_, then we get $P(\Omega_{r})\geq \delta$, *δ* ∈ (0, 1). Notice that for every *ω* ∈ Ω_*r*_, *S*(*τ*_*r*_, *ω*), *I*(*τ*_*r*_, *ω*), *V*(*τ*_*r*_, *ω*) and $e^{m(\tau_{r},w)}$ equals either *r* or $\frac{1}{r}$. Therefore,

$$\begin{array}{c} \begin{array}{cc} W_{1}\left(S\left(0\right),I\left(0\right),V\left(0\right),m\left(0\right)\right)+k_{1}h & \geq E[1_{\Omega_{r}}W_{1}\left(S\left(\tau_{r},h\right),I\left(\tau_{r},h\right),V\left(\tau_{r},h\right),m\left(\tau_{r},h\right)\right)]\\ & \geq \delta [\left(r-1-\text{ln}\;r\right)\wedge \left(\frac{1}{r}-1+\text{ln}\;r\right)\wedge \frac{1}{4}{\left(\text{ln}\;r\right)}^{4}], \end{array} \end{array}$$

where ${\text{1}}_{\Omega_{r}}$ is the indicator function of Ω_*r*_. As *r* → ∞, we have

$$\begin{array}{c} \infty >W_{1}\left(S\left(0\right),I\left(0\right),V\left(0\right),m\left(0\right)\right)+k_{1}h=\infty , \end{array}$$

which creates a paradox. It completes the proof.

**Remark 2.1**. *From Theorem 2.1, there is a unique global solution* (*S*(*t*), *I*(*t*), *V*(*t*), $m(t))\in R_{+}^{3}\times R$. *Hence*

$$\begin{array}{c} \text{d}(S+I+V)\leq [A-\eta (S+I+V)]\text{d}t, \end{array}$$

*and*

$$\begin{array}{c} S\left(t\right)+I\left(t\right)+V\left(t\right)⩽\frac{A}{\eta }+e^{-\eta t}\left(S\left(0\right)+I\left(0\right)+V\left(0\right)-\frac{A}{\eta }\right). \end{array}$$

*If* $S(0)+I(0)+V(0)⩽\frac{A}{\eta }$ , *then* $S(t)+I(t)+V(t)⩽\frac{A}{\eta }$ a.s.. *This suggests that the domain is a positive invariant set. Namely*,

$$\begin{array}{c} \Gamma =\{\left(S,I,V,m\right)\in R_{+}^{3}\times R:S>0,I>0,V>0,S+I+V\leq \frac{A}{\eta }\}. \end{array}$$

## 3 Extinction and persistence

We derive the requirements for when the illness becomes extinct and when it becomes endemic in this section. At the first, we give the definitions

$$\begin{array}{c} R_{0}^{E}=R_{0}+\frac{A\theta }{\sqrt{\pi k}\eta \left(\eta +\gamma +\varepsilon \right)}\;and\;R_{0}^{S}=R_{0}-\frac{A\theta }{\sqrt{\pi k}\eta \left(\eta +\gamma +\varepsilon \right)}. \end{array}$$

From this, it follows that when *m* = 0, the threshold condition for disease extinction and persistence for model Eq (5) is the threshold *R*_0_ for deterministic model disease extinction and persistence.

**Theorem 3.1**. *If* $R_{0}^{E}<1$ *holds, then*

$$\begin{array}{c} \underset{t\to \infty }{\text{lim}}\;\text{sup}\frac{\text{ln}\;I(t)}{t}\leq \left(\eta +\gamma +\varepsilon \right)\left(R_{0}^{E}-1\right)<0\;\text{a}.\text{s}.. \end{array}$$

*I*(*t*) *in system* Eq (5) *is exponentially approaching 0 a.s., that is, the infectiousness of the disease will disappear. Moreover*,

$$\begin{array}{c} \underset{t\to \infty }{\text{lim}}\;S\left(t\right)=\frac{A[\eta (1-g)+\alpha ]}{\eta (\eta +\alpha +p)}=S_{0},\;\underset{t\to \infty }{\text{lim}}V\left(t\right)=\frac{A(\eta g+p)}{\eta (\eta +\alpha +p)}=V_{0},\;\text{a}.\text{s}.. \end{array}$$

**Proof**. Define the following functions:

$$\begin{array}{c} W_{2}=\text{ln}\;I+\frac{\beta }{\eta +p-\frac{\alpha p}{\eta +\alpha }}\left(S+I+\frac{\alpha }{\eta +\alpha }V\right). \end{array}$$

By using Itô’s formula, we get

$$\begin{array}{c} \text{d}W_{2}=LW_{2}\text{d}t, \end{array}$$

where

$$\begin{array}{c} \begin{array}{cc} LW_{2}= & mS-\left(\eta +\gamma +\varepsilon \right)+\frac{\beta [\left(1-g\right)A+\frac{\alpha }{\eta +\alpha }gA]}{\eta +p-\frac{\alpha p}{\eta +\alpha }}-\frac{\beta }{\eta +p-\frac{\alpha p}{\eta +\alpha }}\left(\eta +\varepsilon \right)I\\ ⩽ & \left|m\right|\frac{A}{\eta }-\left(\eta +\gamma +\varepsilon \right)+\frac{\beta [\left(1-g\right)A+\frac{\alpha }{\eta +\alpha }gA]}{\eta +p-\frac{\alpha p}{\eta +\alpha }}\\ = & \left|m\right|\frac{A}{\eta }+\left(\eta +\gamma +\varepsilon \right)\left(R_{0}-1\right). \end{array} \end{array}$$ (7)

Integrating each sides of Eq (7) and dividing by *t* yields

$$\begin{array}{c} \frac{W_{2}\left(t\right)}{t}-\frac{W_{2}\left(0\right)}{t}⩽\left(\eta +\gamma +\varepsilon \right)\left(R_{0}-1\right)+\frac{1}{t}\int_{0}^{t}\frac{A}{\eta }\left|m\left(\tau \right)\right|\text{d}\tau \end{array}$$

Then, combining Eq (4) and taking limits, we get

$$\begin{array}{c} \begin{array}{cc} \underset{t\to \infty }{\text{lim}}\;\text{sup}\frac{\text{ln}\;I(t)}{t}⩽ & \underset{t\to \infty }{\text{lim}}\;\text{sup}\left(\frac{W_{2}\left(t\right)}{t}-\frac{W_{2}\left(0\right)}{t}\right)\\ ⩽ & \left(\eta +\gamma +\varepsilon \right)\left(R_{0}-1\right)+\underset{t\to \infty }{\text{lim}}\frac{1}{t}\int_{0}^{t}\frac{A}{\eta }\left|m\left(\tau \right)\right|\text{d}\tau \\ = & \left(\eta +\gamma +\varepsilon \right)\left(R_{0}+\frac{A\theta }{\sqrt{\pi k}\eta \left(\eta +\gamma +\varepsilon \right)}-1\right)\\ ⩽ & 0, \end{array} \end{array}$$

which hints

$$\begin{array}{c} \underset{t\to \infty }{\text{lim}}\;I\left(t\right)=0\;\text{a}.\text{s}.. \end{array}$$ (8)

From system Eq (5), it can be generated

$$\begin{array}{c} \text{d}(S+I+V)=[A-\eta (S+I+V)-\varepsilon I]\text{d}t. \end{array}$$

Then, using Eq (8), we have

$$\begin{array}{c} \begin{array}{cc} \underset{t\to \infty }{\text{lim}}\left(S\left(t\right)+V\left(t\right)\right) & =\underset{t\to \infty }{\text{lim}}\left\{e^{-\eta t}\left[S\left(0\right)+I\left(0\right)+V\left(0\right)\right]+e^{-\eta t}\int_{0}^{t}\left[A-\varepsilon I\left(u\right)\right]e^{\eta u}\text{d}u-I\left(t\right)\right\}\\ & =\frac{A}{\eta }. \end{array} \end{array}$$ (9)

From the third equation of system Eq (5),

$$\begin{array}{c} \text{d}V\left(t\right)=[gA+p\frac{A}{\eta }-\left(\eta +\alpha +p\right)V\left(t\right)]\text{d}t, \end{array}$$

so then

$$\begin{array}{c} \underset{t\to \infty }{\text{lim}}V\left(t\right)=\frac{A(\eta g+p)}{\eta (\eta +\alpha +p)}=V_{0}\;\text{a}.\text{s}.. \end{array}$$

Thus, from Eq (9), we have

$$\begin{array}{c} \underset{t\to \infty }{\text{lim}}\;S\left(t\right)=\frac{A[\eta (1-g)+\alpha ]}{\eta (\eta +\alpha +p)}=S_{0}\;\text{a}.\text{s}.. \end{array}$$

The proof of the theory is complete.

**Theorem 3.2**. *If* $R_{0}^{S}>1$ *holds, then*

$$\begin{array}{c} \underset{t\to \infty }{\text{lim}}\;\text{inf}\frac{1}{t}\int_{0}^{t}I(\tau )\text{d}\tau ⩾\frac{\eta (\eta +\alpha +p)(\eta +\gamma +\varepsilon )}{\beta (\eta +\varepsilon )(\eta +\alpha )}\left(R_{0}^{S}-1\right),\;\text{a}.\text{s}.. \end{array}$$

**Proof**. From the first line of Eq (7),

$$\begin{array}{c} \begin{array}{cc} L(-W_{2})= & -mS-\frac{\beta [\left(1-g\right)A+\frac{\alpha }{\eta +\alpha }Ag]}{\eta +p-\frac{\alpha p}{\eta +\alpha }}+\left(\eta +\gamma +\varepsilon \right)+\frac{\beta }{\eta +p-\frac{\alpha p}{\eta +\alpha }}\left(\eta +\varepsilon \right)I\\ ⩽ & \left|m\right|\frac{A}{\eta }-\left(\eta +\gamma +\varepsilon \right)\left(R_{0}-1\right)+\frac{\beta }{\eta +p-\frac{\alpha p}{\eta +\alpha }}\left(\eta +\varepsilon \right)I. \end{array} \end{array}$$

Integrating both sides, we have

$$\begin{array}{c} \begin{array}{cc} -\frac{W_{2}\left(t\right)-W_{2}\left(0\right)}{t}⩽ & \frac{1}{t}\int_{0}^{t}\frac{A}{\eta }\left|m\left(\tau \right)\right|\text{d}\tau -\left(\eta +\gamma +\varepsilon \right)\left(R_{0}-1\right)\\ & +\frac{\beta }{\eta +p-\frac{\alpha p}{\eta +\alpha }}\left(\eta +\varepsilon \right)\frac{1}{t}\int_{0}^{t}I(\tau )\text{d}\tau . \end{array} \end{array}$$

Combining Eq (4), we have

$$\begin{array}{c} \begin{array}{cc} \underset{t\to \infty }{\text{lim}}\;\text{inf}\frac{1}{t}\int_{0}^{t}I(\tau )\text{d}\tau ⩾ & \frac{\left(\eta +\gamma +\varepsilon \right)\left(R_{0}-1\right)-\frac{A\theta }{\eta \sqrt{\pi k}}}{\frac{\beta }{\eta +p-\frac{\alpha p}{\eta +\alpha }}\left(\eta +\varepsilon \right)}\\ = & \frac{\eta (\eta +\alpha +p)(\eta +\gamma +\varepsilon )}{\beta (\eta +\varepsilon )(\eta +\alpha )}\left(R_{0}^{S}-1\right). \end{array} \end{array}$$

This completes the proof.

**Remark 3.1**. *By looking at the expression for* $R_{0}^{E}$, *it is clear that* $R_{0}^{E}<1$ *can be inferred to be R*_0_ < 1, *which suggests that the condition for disease extinction in both deterministic and stochastic systems can be united as* $R_{0}^{E}<1$. *Similarly*, $R_{0}^{S}>1$ *can be inferred to be R*_0_ > 1, *which suggests that* $R_{0}^{S}>1$ *can be viewed as a unifying threshold for the prevalence of disease in both deterministic and stochastic systems*.

## 4 Stationary distribution

In deterministic infectious disease models, disease persistence is usually expressed as the stability of endemic equilibrium points. Since there is no endemic equilibrium in the stochastic system Eq (5), we examine the stationary distribution that represents the persistence of disease in this part.

**Lemma 4.1**. [36–38] *For any initial value Z*(0) ∈ Γ, *if there exists a bounded closed domain U*_*δ*_ ∈ Γ with a regular boundary,

$$\begin{array}{c} \underset{t\to \infty }{\text{lim}}\;\text{inf}\frac{1}{t}\int_{0}^{t}P(\tau ,Z\left(0\right),U_{\delta })\text{d}\tau >0\;\text{a}.\text{s}., \end{array}$$

*where* $P(\tau ,Z(0),U_{\delta })$ *is the transition probability of Z*(*t*). *That is to say, the system has at least one ergodic stationary distribution*.

**Theorem 4.1**. *If* $R_{0}^{S}>1$ *holds, system* Eq (5) *exists a stationary distribution π*(⋅).

**Proof**. Using Itô’s formula to system Eq (5), we can have

$$\begin{array}{c} \begin{array}{cc} L(-W_{2})⩽ & \left|m\right|\frac{A}{\eta }-\left(\eta +\gamma +\varepsilon \right)\left(R_{0}-1\right)+\frac{\beta }{\eta +p-\frac{\alpha p}{\eta +\alpha }}\left(\eta +\varepsilon \right)I\\ = & \frac{A\theta }{\sqrt{\pi k}\eta }-\left(\eta +\gamma +\varepsilon \right)\left(R_{0}-1\right)+\left|m\right|\frac{A}{\eta }-\frac{A\theta }{\sqrt{\pi k}\eta }+\frac{\beta }{\eta +p-\frac{\alpha p}{\eta +\alpha }}\left(\eta +\varepsilon \right)I\\ = & -\left(\eta +\gamma +\varepsilon \right)\left(R_{0}^{S}-1\right)+\frac{A}{\eta }\left(|m\right|-\frac{\theta }{\sqrt{\pi k}})+\frac{\beta }{\eta +p-\frac{\alpha p}{\eta +\alpha }}\left(\eta +\varepsilon \right)I, \end{array} \end{array}$$

$$\begin{array}{c} \begin{array}{cc} L(-\text{ln}\;S)= & -\frac{(1-g)A}{S}+\beta I+mI+\left(\eta +p\right)-\frac{\gamma I}{S}-\frac{\alpha V}{S}\\ ⩽ & -\frac{(1-g)A}{S}+\beta I+\left|m\right|\frac{A}{\eta }+\left(\eta +p\right), \end{array} \end{array}$$

$$\begin{array}{c} L\left(-\text{ln}\;V\right)=-\frac{Ag}{V}-\frac{pS}{V}+\left(\eta +\alpha \right)⩽-\frac{Ag}{V}+\left(\eta +\alpha \right), \end{array}$$

$$\begin{array}{c} L\left[-\text{ln}\left(\frac{A}{\eta }-S-I-V\right)\right]=\frac{A-\eta (S+I+V)-\varepsilon I}{\frac{A}{\eta }-S-I-V}⩽\eta -\frac{\varepsilon I}{\frac{A}{\eta }-S-I-V}. \end{array}$$

Denote

$$\begin{array}{c} \bar{W}\left(S,I,V,m\right)=-QW_{2}-\text{ln}\;S-\text{ln}\;V-\text{ln}\left(\frac{A}{\eta }-S-I-V\right)+\frac{m^{2}}{2}, \end{array}$$

and

$$\begin{array}{c} B=3\eta +p+\alpha +\frac{A^{2}}{2k\eta^{2}}+\frac{\theta^{2}}{2}, \end{array}$$

where *Q* is an adequately big and positive constant that fulfills the next inequality:

$$\begin{array}{c} -Q\left(\eta +\gamma +\varepsilon \right)\left(R_{0}^{S}-1\right)+B⩽-2. \end{array}$$

$\bar{W}(S,I,V,m)$ has the lowest value $\bar{W}(S_{0},I_{0},V_{0},m_{0})$ because $\bar{W}(S,I,V,m)\to +\infty$, when (*S*, *I*, *V*, *m*) is near the boundary of Γ. We may therefore construct a non-negative function:

$$\begin{array}{c} W\left(S,I,V,m\right)=\bar{W}\left(S,I,V,m\right)-\bar{W}\left(S_{0},I_{0},V_{0},m_{0}\right). \end{array}$$

Employing Itô’s formula, we can obtain

$$\begin{array}{c} \begin{array}{cc} LW⩽ & -Q\left(\eta +\gamma +\varepsilon \right)\left(R_{0}^{S}-1\right)+Q\frac{A}{\eta }\left(|m\right|-\frac{\theta }{\sqrt{\pi k}})+\frac{Q\beta }{\eta +p-\frac{\alpha p}{\eta +\alpha }}\left(\eta +\varepsilon \right)I-\frac{(1-g)A}{S}\\ & +\beta I+\left|m\right|\frac{A}{\eta }+\left(\eta +p\right)-\frac{Ag}{V}+\left(\eta +\alpha \right)+\eta -\frac{\varepsilon I}{\frac{A}{\eta }-S-I-V}-km^{2}+\frac{\theta^{2}}{2}\\ ⩽ & -Q\left(\eta +\gamma +\varepsilon \right)\left(R_{0}^{S}-1\right)+\left(3\eta +p+\alpha \right)+\frac{A^{2}}{2k\eta^{2}}+\frac{\theta^{2}}{2}+[\frac{Q\beta }{\eta +p-\frac{\alpha p}{\eta +\alpha }}\left(\eta +\varepsilon \right)\\ & +\beta ]I-\frac{(1-g)A}{S}-\frac{Ag}{V}-\frac{\varepsilon I}{\frac{A}{\eta }-S-I-V}-\frac{k}{2}m^{2}+Q\frac{A}{\eta }\left(|m\right|-\frac{\theta }{\sqrt{\pi k}})\\ = & H\left(S,I,V,m\right)+Q\frac{A}{\eta }\left(|m\right|-\frac{\theta }{\sqrt{\pi k}}), \end{array} \end{array}$$

where

$$\begin{array}{c} \begin{array}{cc} H(S,I,V,m)= & -Q\left(\eta +\gamma +\varepsilon \right)\left(R_{0}^{S}-1\right)+\left(3\eta +p+\alpha \right)+\frac{A^{2}}{2k\eta^{2}}+\frac{\theta^{2}}{2}-\frac{(1-g)A}{S}\\ & +\left[\frac{Q\beta }{\eta +p-\frac{\alpha p}{\eta +\alpha }}\left(\eta +\varepsilon \right)+\beta \right]I-\frac{Ag}{V}-\frac{\varepsilon I}{\frac{A}{\eta }-S-I-V}-\frac{k}{2}m^{2}. \end{array} \end{array}$$

Then, define a closed subset of *U*_*δ*_ by

$$\begin{array}{c} U_{\delta }=\{\left(S,I,V,m\right)\in \Gamma |S⩾\delta ,I⩾\delta ,V⩾\delta ,S+I+V⩽\frac{A}{\eta }-\delta^{2},|m|⩽\frac{1}{\delta }\}. \end{array}$$

The following inequalities hold because *δ* is an adequately small constant.

$$\begin{array}{c} -2+[\frac{Q\beta }{\eta +p-\frac{\alpha p}{\eta +\alpha }}\left(\eta +\varepsilon \right)+\beta ]\delta ⩽-1, \end{array}$$

$$\begin{array}{c} -2+\left[\frac{Q\beta }{\eta +p-\frac{\alpha p}{\eta +\alpha }}\left(\eta +\varepsilon \right)+\beta \right]\frac{A}{\eta }-\frac{(1-g)A}{\delta }⩽-1, \end{array}$$

$$\begin{array}{c} -2+\left[\frac{Q\beta }{\eta +p-\frac{\alpha p}{\eta +\alpha }}\left(\eta +\varepsilon \right)+\beta \right]\frac{A}{\eta }-\frac{Ag}{\delta }⩽-1, \end{array}$$

$$\begin{array}{c} -2+\left[\frac{Q\beta }{\eta +p-\frac{\alpha p}{\eta +\alpha }}\left(\eta +\varepsilon \right)+\beta \right]\frac{A}{\eta }-\frac{\varepsilon }{\delta }⩽-1, \end{array}$$

$$\begin{array}{c} -2+\left[\frac{Q\beta }{\eta +p-\frac{\alpha p}{\eta +\alpha }}\left(\eta +\varepsilon \right)+\beta \right]\frac{A}{\eta }-\frac{k}{2\delta^{2}}⩽-1. \end{array}$$

Divide the complement of *U*_*δ*_ into 5 subsets:

$$\begin{array}{c} \begin{array}{cc} U_{1,\delta }^{c} & =\{(S,I,V,m)\in \Gamma |I<\delta \},\\ U_{2,\delta }^{c} & =\{(S,I,V,m)\in \Gamma |S<\delta \},\\ U_{3,\delta }^{c} & =\{(S,I,V,m)\in \Gamma |V<\delta \},\\ U_{4,\delta }^{c} & =\{\left(S,I,V,m\right)\in \Gamma |I⩾\delta ,S+I+V>\frac{A}{\eta }-\delta^{2}\},\\ U_{5,\delta }^{c} & =\{\left(S,I,V,m\right)\in \Gamma |I⩾\delta ,|m|>\frac{1}{\delta }\}. \end{array} \end{array}$$

This gives us the following outcomes.

Case 1. $(S,I,V,m)\in U_{1,\delta }^{c}$, then

$$\begin{array}{c} H\left(S,I,V,m\right)⩽-2+[\frac{Q\beta }{\eta +p-\frac{\alpha p}{\eta +\alpha }}\left(\eta +\varepsilon \right)+\beta ]\delta ⩽-1. \end{array}$$

Case 2. $(S,I,V,m)\in U_{2,\delta }^{c}$, then

$$\begin{array}{c} H\left(S,I,V,m\right)⩽-2+\left[\frac{Q\beta }{\eta +p-\frac{\alpha p}{\eta +\alpha }}\left(\eta +\varepsilon \right)+\beta \right]\frac{A}{\eta }-\frac{(1-g)A}{\delta }⩽-1. \end{array}$$

Case 3. $(S,I,V,m)\in U_{3,\delta }^{c}$, then

$$\begin{array}{c} H\left(S,I,V,m\right)⩽-2+\left[\frac{Q\beta }{\eta +p-\frac{\alpha p}{\eta +\alpha }}\left(\eta +\varepsilon \right)+\beta \right]\frac{A}{\eta }-\frac{Ag}{\delta }⩽-1. \end{array}$$

Case 4. $(S,I,V,m)\in U_{4,\delta }^{c}$, then

$$\begin{array}{c} H\left(S,I,V,m\right)⩽-2+\left[\frac{Q\beta }{\eta +p-\frac{\alpha p}{\eta +\alpha }}\left(\eta +\varepsilon \right)+\beta \right]\frac{A}{\eta }-\frac{\varepsilon }{\delta }⩽-1. \end{array}$$

Case 5. $(S,I,V,m)\in U_{5,\delta }^{c}$, then

$$\begin{array}{c} H\left(S,I,V,m\right)⩽-2+\left[\frac{Q\beta }{\eta +p-\frac{\alpha p}{\eta +\alpha }}\left(\eta +\varepsilon \right)+\beta \right]\frac{A}{\eta }-\frac{k}{2\delta^{2}}⩽-1. \end{array}$$

Given the above five cases, there is a constant *δ* that causes

$$\begin{array}{c} H\left(S,I,V,m\right)⩽-1,\;\forall \left(S,I,V,m\right)\in \Gamma \backslash U_{\delta }. \end{array}$$

In addition, assume that *R* > 0 such that

$$\begin{array}{c} H(S,I,V,m)⩽R<+\infty ,\;\forall (S,I,V,m)\in \Gamma . \end{array}$$

For simplicity, we denote *Z*(*t*) = (*S*(*t*), *I*(*t*), *V*(*t*), *m*(*t*)). For the arbitrary initial value *Z*(0) ∈ Γ, we can have

$$\begin{array}{c} \begin{array}{cc} 0 & ⩽\frac{E[W(Z(t))]}{t}=\frac{E[W(Z(0))]}{t}+\frac{1}{t}\int_{0}^{t}E[LW(Z(\tau ))]\text{d}\tau \\ & ⩽\frac{E[W(Z(0))]}{t}+\frac{1}{t}\int_{0}^{t}E[H(Z(\tau ))]\text{d}\tau +\frac{QA}{\eta }\frac{1}{t}\int_{0}^{t}E\left[\left|m\left(\tau \right)\right|-\frac{\theta }{\sqrt{\pi k}}\right]\text{d}\tau . \end{array} \end{array}$$

By taking the infimum bound for the inequality mentioned above and then associating the results with Eq (4), we get

$$\begin{array}{c} \begin{array}{cc} 0⩽ & \underset{t\to \infty }{\text{lim}}\;\text{inf}\frac{1}{t}\int_{0}^{t}E[H(Z(\tau ))]\text{d}\tau \\ = & \underset{t\to \infty }{\text{lim}}\;\text{inf}\frac{1}{t}\int_{0}^{t}E[H\left(Z\left(\tau \right)\right)1_{\left\{Z\left(\tau \right)\in U_{\delta }\right\}}]\text{d}\tau +\underset{t\to \infty }{\text{lim}}\;\text{inf}\frac{1}{t}\int_{0}^{t}E[H\left(Z\left(\tau \right)\right)1_{\left\{Z\left(\tau \right)\in \Gamma \backslash U_{\delta }\right\}}]\text{d}\tau \\ ⩽ & R\;\underset{t\to \infty }{\text{lim}}\;\text{inf}\frac{1}{t}\int_{0}^{t}P\{Z\left(\tau \right)\in U_{\delta }\}\text{d}\tau -\underset{t\to \infty }{\text{lim}}\;\text{inf}\frac{1}{t}\int_{0}^{t}P\{Z\left(\tau \right)\in \Gamma \backslash U_{\delta }\}\text{d}\tau \\ ⩽ & -1+\left(R+1\right)\underset{t\to \infty }{\text{lim}}\;\text{inf}\frac{1}{t}\int_{0}^{t}P\{Z\left(\tau \right)\in U_{\delta }\}\text{d}\tau , \end{array} \end{array}$$

where $1_{\left\{Z(\tau )\in U_{\delta }\right\}}$ and $1_{\left\{Z(\tau )\in \Gamma \backslash U_{\delta }\right\}}$ are the indicator functions of the set {*Z*(*τ*) ∈ *U*_*δ*_} and {*Z*(*τ*) ∈ Γ\*U*_*δ*_}. This suggests that

$$\begin{array}{c} \underset{t\to \infty }{\text{lim}}\;\text{inf}\frac{1}{t}\int_{0}^{t}P\{Z\left(\tau \right)\in U_{\delta }\}\text{d}\tau ⩾\frac{1}{R+1}. \end{array}$$

Therefore,

$$\begin{array}{c} \underset{t\to \infty }{\text{lim}}\;\text{inf}\frac{1}{t}\int_{0}^{t}P\{\tau ,Z\left(0\right),U_{\delta }\}\text{d}\tau ⩾\frac{1}{R+1}>0,\;\forall Z\left(0\right)\in \Gamma \;\text{a}.\text{s}.. \end{array}$$

Since Γ is invariant under the system Eq (5), the solution (*S*(*t*), *I*(*t*), *V*(*t*), *m*(*t*)) is analysed on Γ. Based on the results in Ref. [37] and from the invariance of Γ and the above inequality, it can be inferred that there exists an invariant probability measure on Γ. Therefore, when $R_{0}^{S}>1$, there exists a stationary distribution on Γ for system Eq (5).

## 5 Density function

It is important to note that the probability density function can reveal a variety of characteristics of the disease dynamics. By transforming the stochastic model Eq (5) into a linearized system, we work on solving for an accurate expression for the probability density function surrounding the quasi-endemic equilibrium. If $R_{0}^{S}>1$ holds, there exists a quasi-endemic equilibrium ${\bar{P}}^{*}=(S^{*},I^{*},V^{*},m^{*})$ satisfying the following equations:

$$\begin{array}{c} \left\{ \begin{array}{c} \left(1-g\right)A-\beta S^{*}I^{*}-m^{*}S^{*}I^{*}-\left(\eta +p\right)S^{*}+\gamma I^{*}+\alpha V^{*}=0,\\ \beta S^{*}I^{*}+m^{*}S^{*}I^{*}-\left(\eta +\gamma +\varepsilon \right)I^{*}=0,\\ Ag+pS^{*}-\left(\eta +\alpha \right)V^{*}=0,\\ -km^{*}=0. \end{array}\right. \end{array}$$

When stochastic factors are not taken into account in the model, the deterministic model Eq (2) has the identical endemic equilibrium as the quasi-endemic equilibrium. Let *u*_1_ = *S* − S*, *u*_2_ = *I* − I*, *u*_3_ = *V* − V*, *u*_4_ = *m* − m*, and we obtain the linearized system

$$\begin{array}{c} \left\{ \begin{array}{cc} \text{d}u_{1}= & (-a_{11}u_{1}-a_{12}u_{2}+a_{13}u_{3}-a_{14}u_{4})\text{d}t,\\ \text{d}u_{2}= & (a_{21}u_{1}+a_{14}u_{4})\text{d}t,\\ \text{d}u_{3}= & (a_{31}u_{1}-a_{33}u_{3})\text{d}t,\\ \text{d}u_{4}= & -a_{44}u_{4}\text{d}t+\theta \text{d}B\left(t\right), \end{array}\right. \end{array}$$ (10)

where

$$\begin{array}{c} \begin{array}{cc} a_{11} & =\beta I^{*}+\eta +p,a_{12}=\beta S^{*}-\gamma =\eta +\varepsilon >0,a_{13}=\alpha ,a_{14}=S^{*}I^{*},a_{21}=\beta I^{*},a_{31}=p,\\ a_{33} & =\eta +\alpha ,a_{44}=k. \end{array} \end{array}$$

**Theorem 5.1**. *If* $R_{0}^{S}>1$, *the solution* (*u*_1_, *u*_2_, *u*_3_, *u*_4_) *of system* Eq (10) *obeys the normal probability density function* Φ(*u*_1_, *u*_2_, *u*_3_, *u*_4_), *and the form of* Φ(*u*_1_, *u*_2_, *u*_3_, *u*_4_) *is as follows*:

$$\begin{array}{c} \Phi \left(u_{1},u_{2},u_{3},u_{4}\right)={\left(2\pi \right)}^{-2}|\Sigma {|}^{-\frac{1}{2}}e^{-\frac{1}{2}\left(u_{1},u_{2,},u_{3},u_{4}\right)\Sigma^{-1}{\left(u_{1},u_{2},u_{3},u_{4}\right)}^{T}}, \end{array}$$

*where*

$$\begin{array}{c} \Sigma ={\left(m_{1}\theta \right)}^{2}{\left(M_{1}J_{3}J_{2}J_{1}\right)}^{-1}\Sigma_{1}{\left[{\left(M_{1}J_{3}J_{2}J_{1}\right)}^{-1}\right]}^{T}, \end{array}$$

*and*

$$\begin{array}{c} J_{1}=(\begin{array}{cccc} 0 & 0 & 0 & 1\\ 1 & 0 & 0 & 0\\ 0 & 1 & 0 & 0\\ 0 & 0 & 1 & 0 \end{array}),J_{2}=(\begin{array}{cccc} 1 & 0 & 0 & 0\\ 0 & 1 & 0 & 0\\ 0 & 1 & 1 & 0\\ 0 & 0 & 0 & 1 \end{array}),J_{3}=(\begin{array}{cccc} 1 & 0 & 0 & 0\\ 0 & 1 & 0 & 0\\ 0 & 0 & 1 & 0\\ 0 & 0 & \frac{-a_{31}}{a_{12}+a_{21}-a_{11}} & 1 \end{array}), \end{array}$$

$$\begin{array}{c} M_{1}=(\begin{array}{cccc} m_{1} & m_{2} & m_{3} & m_{4}\\ 0 & a_{4}a_{7} & a_{7}\left(a_{5}+a_{8}\right) & a_{7}a_{6}+a_{8}^{2}\\ 0 & 0 & a_{7} & a_{8}\\ 0 & 0 & 0 & 1 \end{array}), \end{array}$$

$$\begin{array}{c} \Sigma_{1}=(\begin{array}{cccc} \frac{c_{2}c_{3}-c_{1}c_{4}}{2(c_{1}c_{2}c_{3}-c_{3}^{2}-c_{1}^{2}c_{4})} & 0 & \frac{-c_{3}}{2(c_{1}c_{2}c_{3}-c_{3}^{2}-c_{1}^{2}c_{4})} & 0\\ 0 & \frac{c_{3}}{2(c_{1}c_{2}c_{3}-c_{3}^{2}-c_{1}^{2}c_{4})} & 0 & \frac{-c_{1}}{2(c_{1}c_{2}c_{3}-c_{3}^{2}-c_{1}^{2}c_{4})}\\ \frac{-c_{3}}{2(c_{1}c_{2}c_{3}-c_{3}^{2}-c_{1}^{2}c_{4})} & 0 & \frac{c_{1}}{2(c_{1}c_{2}c_{3}-c_{3}^{2}-c_{1}^{2}c_{4})} & 0\\ 0 & \frac{-c_{1}}{2(c_{1}c_{2}c_{3}-c_{3}^{2}-c_{1}^{2}c_{4})} & 0 & \frac{c_{1}c_{2}-c_{3}}{2(c_{1}c_{2}c_{3}-c_{3}^{2}-c_{1}^{2}c_{4})} \end{array}), \end{array}$$

*where*

$$\begin{array}{c} m_{1}=a_{2}a_{4}a_{7},m_{2}=a_{4}a_{7}\left(a_{3}+a_{5}+a_{8}\right),m_{3}=a_{5}a_{7}\left(a_{4}+a_{5}+a_{8}\right)+a_{7}\left(a_{6}a_{7}+a_{8}^{2}\right), \end{array}$$

$$\begin{array}{c} m_{4}=a_{6}a_{7}\left(a_{4}+a_{5}+a_{8}\right)+a_{8}\left(a_{6}a_{7}+a_{8}^{2}\right),a_{2}=-a_{14},a_{3}=a_{12}-a_{11},a_{4}=a_{12} \end{array}$$

$$\begin{array}{c} +a_{21}-a_{11},a_{5}=\frac{a_{13}a_{31}}{a_{12}+a_{21}-a_{11}}-a_{12},a_{6}=a_{13},a_{7}=\left(a_{12}+a_{8}\right)\frac{a_{31}}{a_{12}+a_{1}-a_{11}}, \end{array}$$

$$\begin{array}{c} a_{8}=\frac{-a_{13}a_{31}}{a_{12}+a_{21}-a_{11}}-a_{33},b_{1}=a_{11}+a_{33},b_{2}=a_{12}a_{21}+a_{11}a_{33}-a_{13}a_{31}, \end{array}$$

$$\begin{array}{c} b_{3}=a_{12}a_{21}a_{33},c_{1}=b_{1}+a_{44},c_{2}=b_{2}+a_{44}b_{1},c_{3}=b_{3}+a_{44}b_{2},c_{4}=a_{44}b_{3}. \end{array}$$

**Proof**. Firstly, let d*u* = *Au*d*t* + *G*d*B*(*t*), the matrix form of system Eq (10) is obtained:

$$\begin{array}{c} \text{d}u=(\begin{array}{cccc} -a_{11} & -a_{12} & a_{13} & -a_{14}\\ a_{21} & 0 & 0 & a_{14}\\ a_{31} & 0 & -a_{33} & 0\\ 0 & 0 & 0 & -a_{44} \end{array})u\text{d}t+(\begin{array}{cccc} 0 & 0 & 0 & 0\\ 0 & 0 & 0 & 0\\ 0 & 0 & 0 & 0\\ 0 & 0 & 0 & \theta \end{array})\text{d}B\left(t\right). \end{array}$$

By calculating the polynomial equation for the eigenvalues λ of the matrix *A*, one obtains

$$\begin{array}{c} \varphi_{A}\left(\lambda \right)=\left(\lambda +a_{44}\right)\left(\lambda^{3}+b_{1}\lambda^{2}+b_{2}\lambda +b_{3}\right), \end{array}$$

where *b*_1_ = *a*_11_ + *a*_33_, *b*_2_ = *a*_12_*a*_21_ + *a*_11_*a*_33_ − *a*_13_*a*_31_, *b*_3_ = *a*_12_*a*_21_*a*_33_. The characteristic roots of *φ*_*A*_(λ) can be obtained from λ_1_ = −*a*_44_ and λ^3^ + *b*_1_λ^2^ + *b*_2_λ + *b*_3_ = 0. Then the characteristic roots all contain negative real parts as a result of *b*_1_ > 0, *b*_2_ > 0, *b*_3_ > 0 and *b*_1_*b*_2_ − *b*_3_ > 0. It is evident that *A* is a Hurwitz matrix. According to Ref. [39], the density function Φ(*u*_1_, *u*_2_, *u*_3_, *u*_4_) of system Eq (10) can be represented as the following Fokker-Planck equation:

$$\begin{array}{c} -\frac{\theta^{2}}{2}\frac{\partial^{2}}{\partial u_{4}^{2}}\Phi +\frac{\partial }{\partial u_{4}}\left[\left(-a_{44}u_{4}\right)\Phi \right]+\frac{\partial }{\partial u_{1}}\left[\left(-a_{11}u_{1}-a_{12}u_{2}+a_{13}u_{3}-a_{14}u_{4}\right)\Phi \right] \end{array}$$

$$\begin{array}{c} +\frac{\partial }{\partial u_{2}}\left[\left(a_{21}u_{1}+a_{14}u_{4}\right)\Phi \right]+\frac{\partial }{\partial u_{3}}\left[\left(a_{31}u_{1}-a_{33}u_{3}\right)\Phi \right]=0, \end{array}$$

which may be approximately expressed as the following Gaussian distribution:

$$\begin{array}{c} \Phi \left(u\right)=c\;\text{exp}\left\{-\frac{1}{2}uDu^{T}\right\}. \end{array}$$

Here *D* is a real symmetric matrix fulfilling *DG*^2^*D* + *A*^*T*^*D* + *DA* = 0. Then suppose that it is positive definite and *D*^−1^ = Σ, we have

$$\begin{array}{c} G^{2}+A\Sigma +\Sigma A^{T}=0. \end{array}$$ (11)

Then prove that Σ is positive definite. Let

$$\begin{array}{c} J_{1}=(\begin{array}{cccc} 0 & 0 & 0 & 1\\ 1 & 0 & 0 & 0\\ 0 & 1 & 0 & 0\\ 0 & 0 & 1 & 0 \end{array}), \end{array}$$

then

$$\begin{array}{c} A_{1}=J_{1}AJ_{1}^{-1}=(\begin{array}{cccc} -a_{44} & 0 & 0 & 0\\ -a_{14} & -a_{11} & -a_{12} & a_{13}\\ a_{14} & a_{21} & 0 & 0\\ 0 & a_{31} & 0 & -a_{33} \end{array}). \end{array}$$

Next, let

$$\begin{array}{c} J_{2}=(\begin{array}{cccc} 1 & 0 & 0 & 0\\ 0 & 1 & 0 & 0\\ 0 & 1 & 1 & 0\\ 0 & 0 & 0 & 1 \end{array}), \end{array}$$

we get

$$\begin{array}{c} A_{2}=J_{2}A_{1}J_{2}^{-1}=(\begin{array}{cccc} -a_{44} & 0 & 0 & 0\\ -a_{14} & -a_{11}+a_{12} & -a_{12} & a_{13}\\ 0 & -a_{11}+a_{12}+a_{21} & -a_{12} & a_{13}\\ 0 & a_{31} & 0 & -a_{33} \end{array}). \end{array}$$

Note that

$$\begin{array}{c} J_{3}=(\begin{array}{cccc} 1 & 0 & 0 & 0\\ 0 & 1 & 0 & 0\\ 0 & 0 & 1 & 0\\ 0 & 0 & \frac{-a_{31}}{a_{12}+a_{21}-a_{11}} & 1 \end{array}), \end{array}$$

by calculations, we have

$$\begin{array}{c} A_{3}=J_{3}A_{2}J_{3}^{-1}=(\begin{array}{cccc} a_{1} & 0 & 0 & 0\\ a_{2} & a_{3} & a_{5} & a_{6}\\ 0 & a_{4} & a_{5} & a_{6}\\ 0 & 0 & a_{7} & a_{8} \end{array}), \end{array}$$

where *a*_1_ = −*a*_44_, *a*_2_ = −*a*_14_, *a*_3_ = −*a*_11_ + *a*_12_, *a*_4_ = −*a*_11_ + *a*_12_ + *a*_21_, *a*_5_ = −*a*_12_ $+\frac{a_{13}a_{31}}{a_{12}+a_{21}-a_{11}}$, $a_{6}=a_{13},a_{7}=\frac{(a_{12}-a_{33})a_{31}}{a_{12}+a_{21}-a_{11}}-\frac{a_{13}a_{31}^{2}}{{\left(a_{12}+a_{21}-a_{11}\right)}^{2}},a_{8}=\frac{-a_{13}a_{31}}{a_{12}+a_{21}-a_{11}}-a_{33}$. The transformation matrix *M*_1_ is obtained by following the procedure in Ref. [40],

$$\begin{array}{c} M_{1}=(\begin{array}{cccc} m_{1} & m_{2} & m_{3} & m_{4}\\ 0 & a_{4}a_{7} & a_{7}\left(a_{5}+a_{8}\right) & a_{7}a_{6}+a_{8}^{2}\\ 0 & 0 & a_{7} & a_{8}\\ 0 & 0 & 0 & 1 \end{array}), \end{array}$$

where $m_{1}=a_{2}a_{4}a_{7},m_{2}=a_{4}a_{7}(a_{3}+a_{5}+a_{8}),m_{3}=a_{5}a_{7}(a_{4}+a_{5}+a_{8})+a_{7}(a_{6}a_{7}+a_{8}^{2})$, $m_{4}=a_{6}a_{7}(a_{4}+a_{5}+a_{8})+a_{8}(a_{6}a_{7}+a_{8}^{2})$. Then, by means of the standard *R*_1_ transformation matrix, one obtains

$$\begin{array}{c} B=M_{1}A_{3}{M_{1}}^{-1}=(\begin{array}{cccc} -c_{1} & -c_{2} & -c_{3} & -c_{4}\\ 1 & 0 & 0 & 0\\ 0 & 1 & 0 & 0\\ 0 & 0 & 1 & 0 \end{array}), \end{array}$$

where *c*_1_ = *b*_1_ + *a*_44_, *c*_2_ = *b*_2_ + *a*_44_*b*_1_, *c*_3_ = *b*_3_ + *a*_44_*b*_2_, *c*_4_ = *a*_44_*b*_3_. Then, Eq (11) may be changed into the form shown below:

$$\begin{array}{c} \left(M_{1}J_{3}J_{2}J_{1}\right)G^{2}{\left(M_{1}J_{3}J_{2}J_{1}\right)}^{T}+\left(M_{1}J_{3}J_{2}J_{1}\right)A{\left(M_{1}J_{3}J_{2}J_{1}\right)}^{-1}\left(M_{1}J_{3}J_{2}J_{1}\right)\Sigma {\left(M_{1}J_{3}J_{2}J_{1}\right)}^{T} \end{array}$$

$$\begin{array}{c} +\left(M_{1}J_{3}J_{2}J_{1}\right)\Sigma (M_{1}J_{3}J_{2}J_{1}{)}^{T}{\left[\left(M_{1}J_{3}J_{2}J_{1}\right)A{\left(M_{1}J_{3}J_{2}J_{1}\right)}^{-1}\right]}^{T}=0, \end{array}$$

which can be expressed as

$$\begin{array}{c} G_{0}^{2}+B\Sigma_{1}+\Sigma_{1}B^{T}=0, \end{array}$$

where $\Sigma_{1}=\frac{1}{m_{1}^{2}\theta^{2}}(M_{1}J_{3}J_{2}J_{1})\Sigma (M_{1}J_{3}J_{2}J_{1}{)}^{T}$. Using the Lemma 2.3 in Ref. [40], the form of Σ_1_ can be expressed as follows:

$$\begin{array}{c} \Sigma_{1}=(\begin{array}{cccc} \frac{c_{2}c_{3}-c_{1}c_{4}}{2(c_{1}c_{2}c_{3}-c_{3}^{2}-c_{1}^{2}c_{4})} & 0 & \frac{-c_{3}}{2(c_{1}c_{2}c_{3}-c_{3}^{2}-c_{1}^{2}c_{4})} & 0\\ 0 & \frac{c_{3}}{2(c_{1}c_{2}c_{3}-c_{3}^{2}-c_{1}^{2}c_{4})} & 0 & \frac{-c_{1}}{2(c_{1}c_{2}c_{3}-c_{3}^{2}-c_{1}^{2}c_{4})}\\ \frac{-c_{3}}{2(c_{1}c_{2}c_{3}-c_{3}^{2}-c_{1}^{2}c_{4})} & 0 & \frac{c_{1}}{2(c_{1}c_{2}c_{3}-c_{3}^{2}-c_{1}^{2}c_{4})} & 0\\ 0 & \frac{-c_{1}}{2(c_{1}c_{2}c_{3}-c_{3}^{2}-c_{1}^{2}c_{4})} & 0 & \frac{c_{1}c_{2}-c_{3}}{2(c_{1}c_{2}c_{3}-c_{3}^{2}-c_{1}^{2}c_{4})} \end{array}). \end{array}$$

Moreover, from *b*_1_ > 0, *b*_2_ > 0, *b*_3_ > 0, *a*_44_ > 0 and *b*_1_*b*_2_ − *b*_3_ > 0, it can be inferred that *c*_1_ > 0, *c*_3_ > 0, *c*_4_ > 0 and $c_{1}c_{2}c_{3}-c_{3}^{2}-c_{1}^{2}c_{4}=(b_{1}+a_{44})(b_{1}b_{2}-b_{3})a_{44}^{2}+b_{3}(b_{1}b_{2}-b_{3})+b_{2}(b_{1}b_{2}-b_{3})a_{44}>0$. As a result, the matrix Σ_1_ is positive definite. It is possible to determine the precise expression of Σ, which is the positive definite matrix Σ = (*m*_1_*θ*)^2^(*M*_1_*J*_3_*J*_2_*J*_1_)^−1^Σ_1_[(*M*_1_*J*_3_*J*_2_*J*_1_)^−1^]^*T*^. This proof is complete.

**Remark 5.1**. *By Theorem 5.1, we know that the solution* (*S*(*t*), *I*(*t*), *V*(*t*)) *of the system* Eq (5) *obeys the normal density function* $\Phi (S,I,V)∼N((S^{*},I^{*},V^{*}{)}^{T},\Sigma^{\left(3\right)})$. *Here, we define* Σ^(3)^ *as the third-order principal minor of* Σ. *Hence, S*(*t*), *I*(*t*) *and V*(*t*) *will each converge to the marginal density functions*:

$$\begin{array}{c} \Phi_{S}\left(S\right)=\frac{1}{\sqrt{2\pi }\varphi_{1}}e^{-\frac{{\left(S-S^{*}\right)}^{2}}{2\varphi_{1}^{2}}},\;\Phi_{I}\left(I\right)=\frac{1}{\sqrt{2\pi }\varphi_{2}}e^{-\frac{{\left(I-I^{*}\right)}^{2}}{2\varphi_{2}^{2}}},\;\Phi_{V}\left(V\right)=\frac{1}{\sqrt{2\pi }\varphi_{3}}e^{-\frac{{\left(V-V^{*}\right)}^{2}}{2\varphi_{3}^{2}}}, \end{array}$$

*where* $\varphi_{i}^{2}$ *is the element in row i, column i on* Σ. *Namely, S*(*t*), *I*(*t*) *and V*(*t*) *will converge to the marginal distributions* $N(S^{*},\varphi_{1}^{2})$, $N(I^{*},\varphi_{2}^{2})$ *and* $N(V^{*},\varphi_{3}^{2})$, *respectively*.

## 6 Numerical simulation

In this section, various numerical simulations are provided in order to verify the scientific results. Next, by employing the Milstein method to numerically simulate the model, the correctness of the conclusions is demonstrated. The model Eq (5) is discretized, and the matching discretization model is obtained as follows:

$$\begin{array}{c} \left\{ \begin{array}{cc} S_{i+1}= & S_{i}+\left[\left(1-g\right)A-\left(\beta +m_{i}\right)S_{i}I_{i}-\left(\eta +p\right)S_{i}+\gamma I_{i}+\alpha V_{i}\right]\Delta t,\\ I_{i+1}= & I_{i}+\left[\left(\beta +m_{i}\right)S_{i}I_{i}-\left(\eta +\gamma +\varepsilon \right)I_{i}\right]\Delta t,\\ V_{i+1}= & V_{i}+\left[Ag+pS_{i}-\left(\eta +\alpha \right)V_{i}\right]\Delta t,\\ m_{i+1}= & m_{i}-km_{i}\Delta t+\theta h_{i}\sqrt{\Delta t}+\frac{\theta^{2}}{2}\left(h_{i}^{2}-1\right)\Delta t. \end{array}\right. \end{array}$$

Here Δ*t* is the time interval and takes the value of Δ*t* = 1, and *h*_*i*_ is a random variable that follows a standard normal distribution.

**Fig 1:**
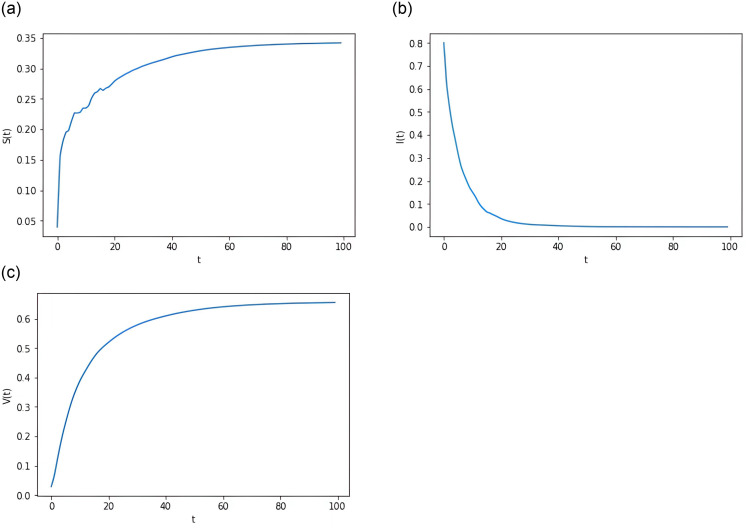
Computer simulations of *S*(*t*), *I*(*t*) and *V*(*t*) for system Eq (5) in case of disease extinction.

**Example 6.1** Assume the parameters *k* = 0.65, *θ* = 0.14 and the starting points in the below examples are all (*S*(0), *I*(0), *V*(0), *m*(0)) = (0.04, 0.8, 0.03, −0.02). Other parameters are as in Group 1 of Table 1. Note that $R_{0}^{E}\approx 0.7697<1$ makes the condition of Theorem 3.1 hold. This shows that *I*(*t*) has an exponential tendency to go to zero with probability 1. Then, as evidence for our findings, we present Fig 1.

**Table 1:** Parameter values.

Group1				Group2			
Parameter	Value	Parameter	Value	Parameter	Value	Parameter	Value
*A*	0.05 [Assumed]	*β*	0.5 [9]	*A*	0.5 [20]	*β*	0.9 [Assumed]
*g*	0.6 [Assumed]	*γ*	0.2 [9]	*g*	0.8 [20]	*γ*	0.3 [20]
*η*	0.05 [Assumed]	*α*	0.1 [9]	*η*	0.1 [20]	*α*	0.2 [20]
*p*	0.2 [Assumed]	*ε*	0.1 [Assumed]	*p*	0.6 [20]	*ε*	0.15 [Assumed]

**Fig 2:**
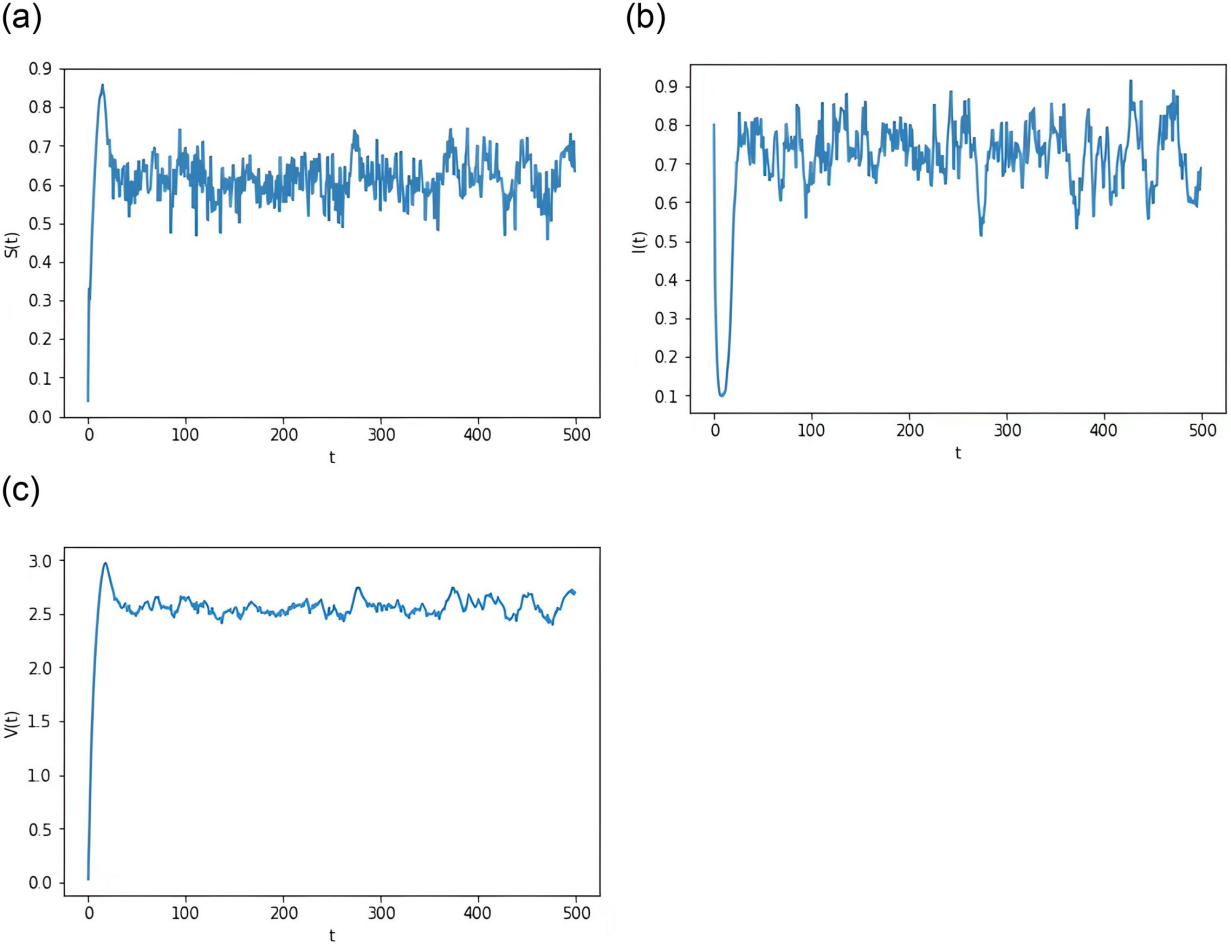
Computer simulations of *S*(*t*), *I*(*t*) and *V*(*t*) for system Eq (5) in the presence of disease persistence.

**Example 6.2** Assume the parameters *k* = 0.7, *θ* = 0.1, and other parameters are as in Group 2 of Table 1. Note that $R_{0}^{S}\approx 1.3870>1$ satisfies the requirement of Theorem 3.2. In other words, the illness is going to prevail. Then, as evidence for our findings, we present Fig 2.

**Fig 3:**
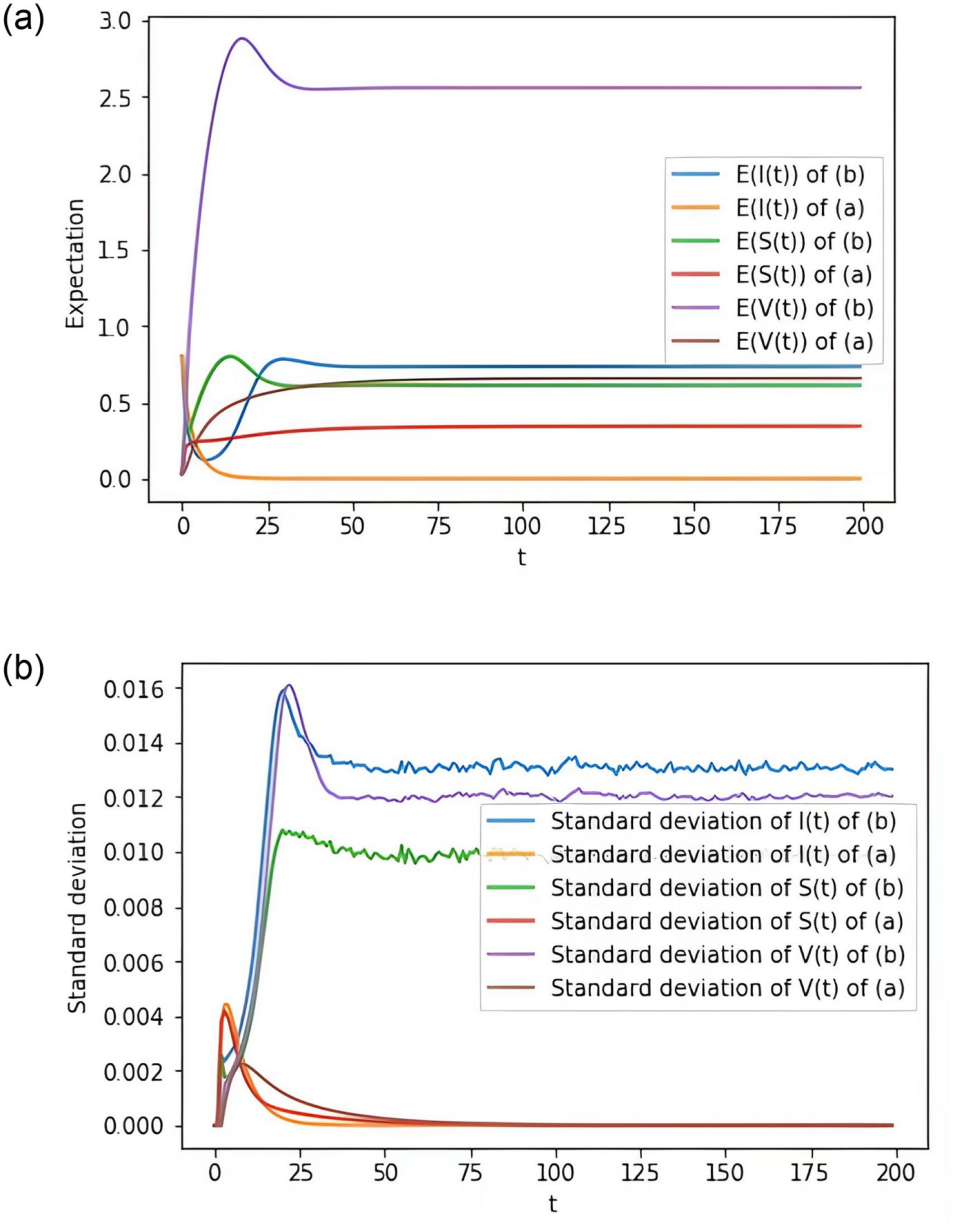
Computer simulations of expectation and standard deviation for the model Eq (5).

**Example 6.3** Assume that the parameters in group (a) take the same values as in Example 6.1 and that the parameters in group (b) take the same values as in Example 6.2. Through 10,000 stochastic simulations, we can obtain Fig 3 representing the expectation and standard deviation of *S*(*t*), *I*(*t*) and *V*(*t*). It shows that the disease will quickly die out in case (a), while the disease will become endemic in case (b).

**Fig 4:**
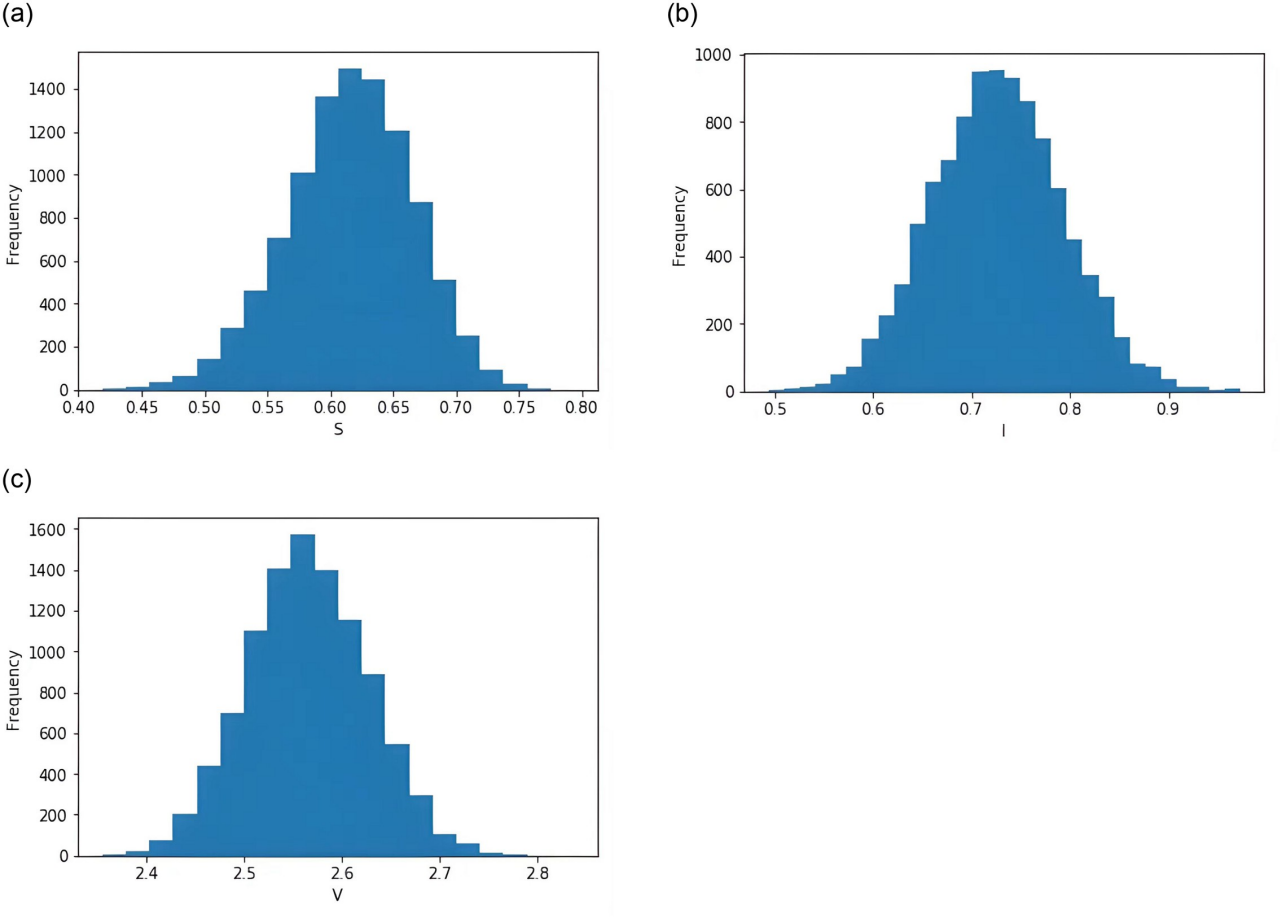
Computer simulations of the histograms of frequencies for the model Eq (5).

**Example 6.4** It is assumed that these parameters are selected with the identical values as in Example 6.2. Based on 10,000 stochastic simulations, the ergodic stationary distribution is proved in Fig 4. It is obviously that the value on the histogram surround P* = (S*, I*, V*) ≈ (0.6111, 0.7333, 2.5555) of the deterministic model. The solution (*S*(*t*), *I*(*t*), *V*(*t*), *m*(*t*)) of system Eq (5) obeys the normal density function $\Phi (S,I,V,m)∼N((0.6111,0.7333,2.555,0{)}^{T},\Sigma )$. The matrix Σ is represented as

$$\begin{array}{c} \Sigma =1\times 10^{-2}(\begin{array}{cccc} 0.0706 & -0.1426 & 0.0410 & -0.2055\\ -0.1426 & 0.2391 & -0.1016 & 0.2590\\ 0.0410 & -0.1016 & 0.0870 & 0.0386\\ -0.2055 & 0.2590 & 0.0386 & 0.9124 \end{array}), \end{array}$$

from which the following three marginal density functions are inferred

$$\begin{array}{c} \Phi_{S}\left(S\right)=15.01332e^{-708.11447{\left(S-0.6111\right)}^{2}}, \end{array}$$

$$\begin{array}{c} \Phi_{I}\left(I\right)=8.15787e^{-209.07572{\left(I-0.7333\right)}^{2}}, \end{array}$$

$$\begin{array}{c} \Phi_{V}\left(V\right)=13.52366e^{-574.56414{\left(V-2.5555\right)}^{2}}. \end{array}$$

**Example 6.5** Consider the corresponding discretized deterministic SIVS model

$$\begin{array}{c} \left\{ \begin{array}{cc} S_{i+1}= & S_{i}+\left[\left(1-g\right)A-\beta S_{i}I_{i}-\left(\eta +p\right)S_{i}+\gamma I_{i}+\alpha V_{i}\right]\Delta t,\\ I_{i+1}= & I_{i}+\left[\beta S_{i}I_{i}-\left(\eta +\gamma +\varepsilon \right)I_{i}\right]\Delta t,\\ V_{i+1}= & V_{i}+\left[Ag+pS_{i}-\left(\eta +\alpha \right)V_{i}\right]\Delta t. \end{array}\right. \end{array}$$

**Fig 5:**
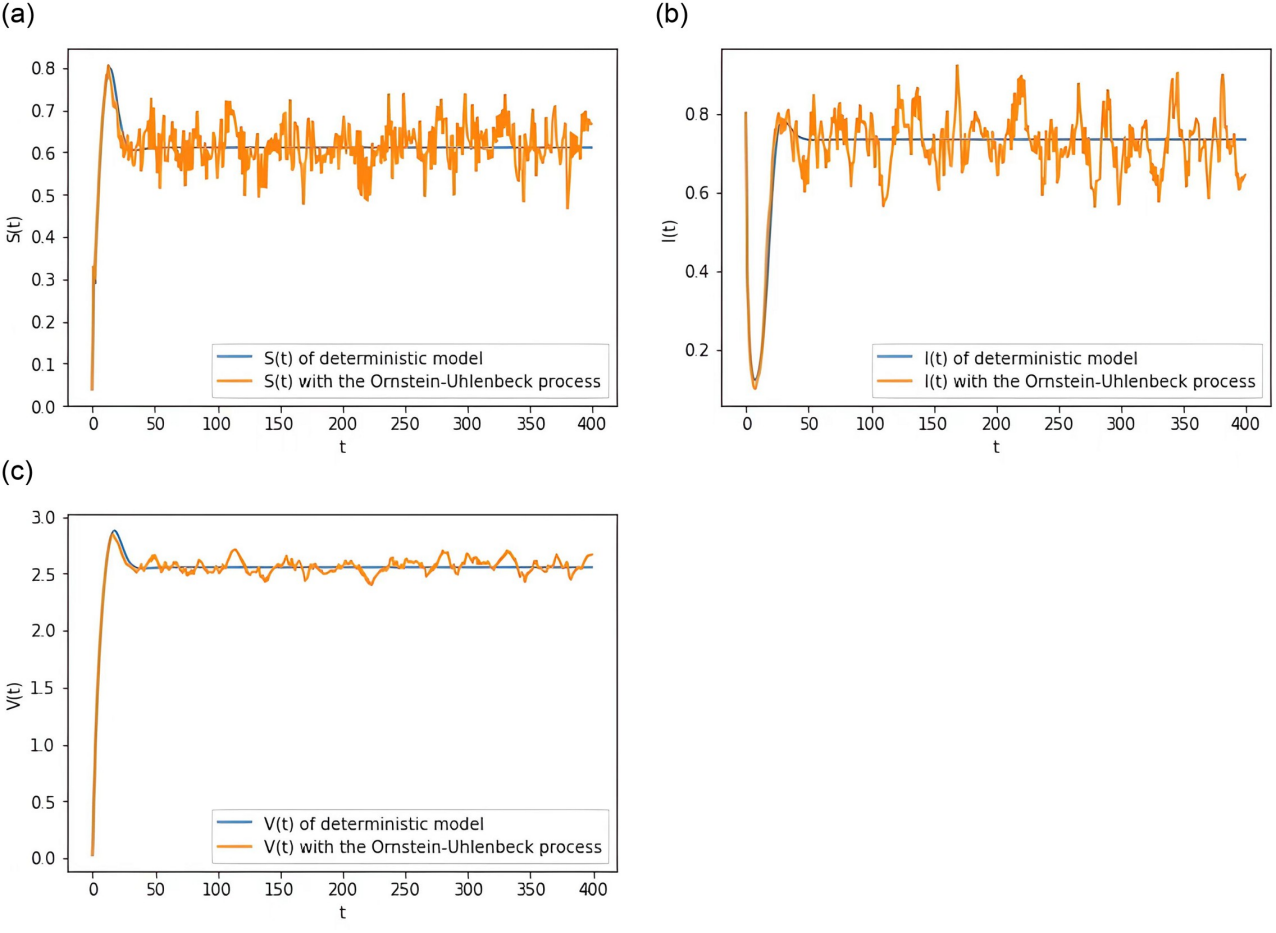
Comparison plot between the stochastic model Eq (5) with the mean-reverting process and deterministic model Eq (1).

Assume that the parameters take the same values as in Example 6.2. The mean-reverting process is substituted into the deterministic SIVS model to transform it into the stochastic SIVS model. Then we draw a comparison chart between deterministic SIVS model and stochastic SIVS model in Fig 5. It shows that the stochastic SIVS model that includes the mean-reverting process fluctuates around the deterministic model.

**Fig 6:**
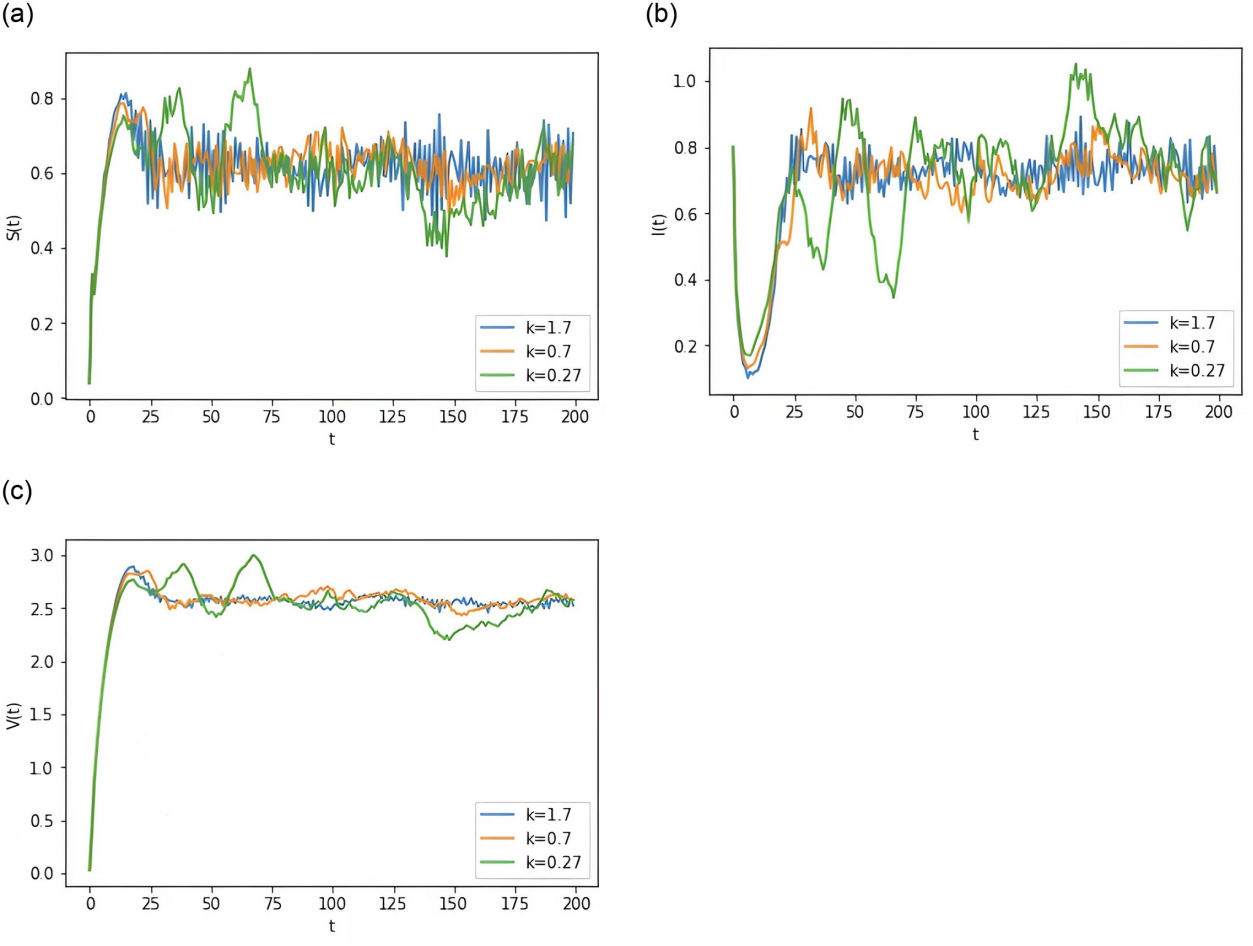
Computer simulations for different values of *k* for the model Eq (5).

**Example 6.6** Assume the parameters *θ* = 0.1, and other parameters are as in Group 2 of Table 1. To study the effect of *k* size on disease progression. As can be seen in Fig 6, the fluctuation of the disease becomes smaller as *k* increases.

**Fig 7:**
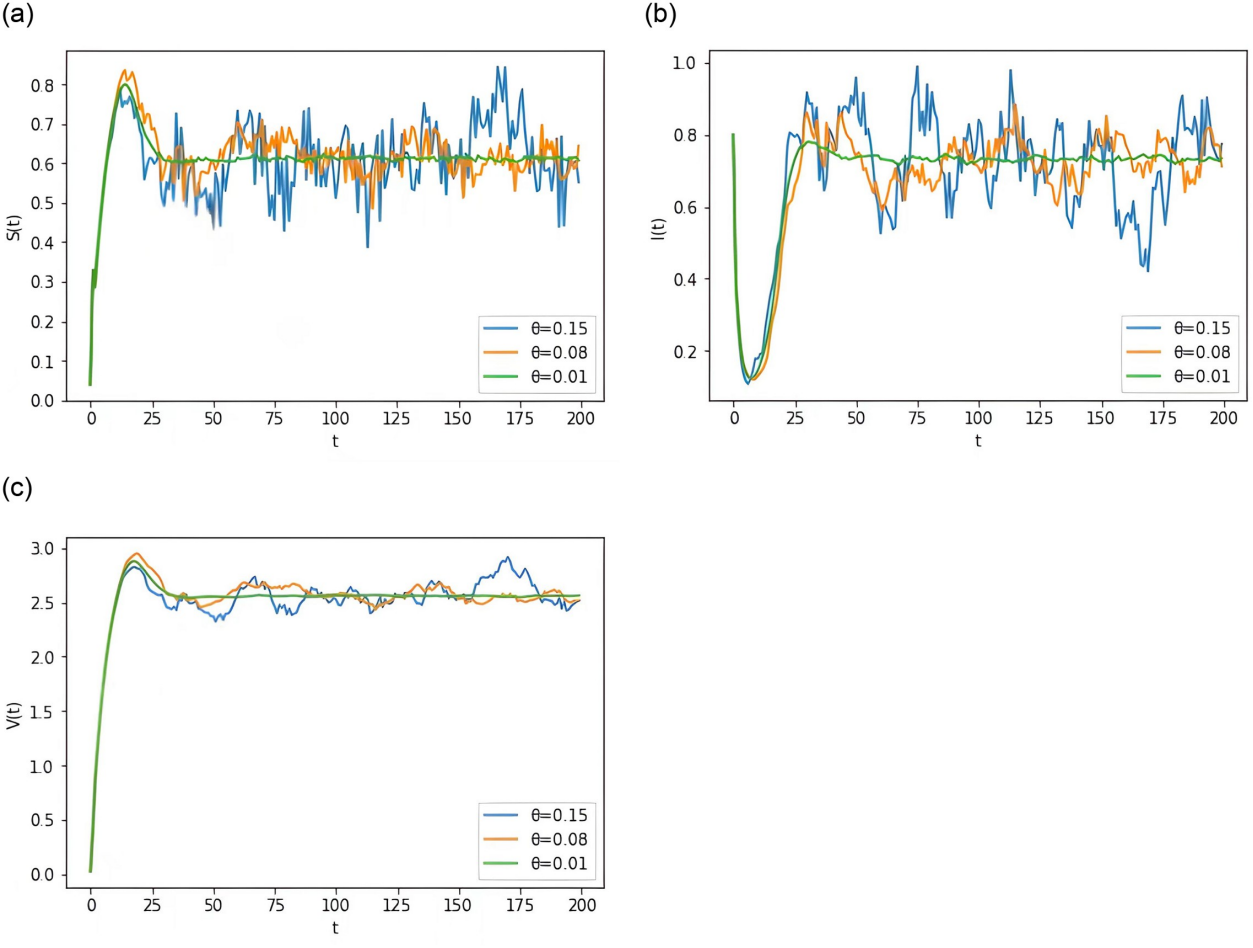
Computer simulations for different values of *θ* for the model Eq (5).

**Example 6.7** Assume the parameters *k* = 0.7, and other parameters are as in Group 2 of Table 1. To study the effect of *θ* size on disease progression. As can be seen in Fig 7, the fluctuation of the disease becomes larger as *θ* increases.

## 7 Conclusion

We demonstrate necessity and rationale of introducing the Ornstein-Uhlenbeck process by contrasting it with white Gaussian noise. Considering the theoretical results obtained in Ref. [11], we concentrate on a stochastic model with the mean-reverting process. Instead of the typical linear function with white noise, the parameter *β* is designed to fulfill the mean-reversion process. By doing this, the issue that Var[β(t)] may become infinite as the time interval becomes smaller is avoided. In this paper, in the situation where the unique global positive solution to the system Eq (5) exists, the conditions for disappearance and persistence of the illness are gained, i.e., when $R_{0}^{E}<1$, the illness tends to extinction; when $R_{0}^{S}>1$, the illness becomes epidemic. Further, the system Eq (5) exists a stationary distribution when $R_{0}^{S}>1$, indicating that the illness will likely end up becoming an epidemic that lasts for a long time. To further study the infectious disease dynamics, by resolving the related equations, the probability density function surrounding the quasi-endemic equilibrium is explored, which reveals many dynamical features. The final step is to demonstrate theoretical results through numerical simulation. Through numerical simulations, we find an interesting conclusion: smaller regression rates or larger fluctuation intensities make the stochastic system more volatile.

In addition, a uniform threshold for disease extinction and prevalence in stochastic systems remains difficult to obtain due to limitations of existing mathematical methods. We expect that this problem can be solved in future studies.
